# Oxygen in Therapeutics

**Published:** 1887-11

**Authors:** 


### BOOK NOTICES AND REVIEWS.

Oxygen in Therapeutics. By C. E. Ehinger, M. D. Chicago: W. A. Chatterton & Co., 1887.

This monograph, as its name implies, is a review of the uses of oxygen in the treatment of disease. It is a most excellent essay, giving a summary of all that is known upon the subject. The methods of making oxygen and purifying it, the method of applying it, the diseases for which it is useful, and a report of cases actually treated and cured, are all given clearly and yet concisely. Whoever wishes to get a comprehensive idea of how oxygen is used in medicine, can find in this book all he requires to know.

Oxygen has been identified mostly with quackery. But this book, by its dignity, temperance, and truthfulness of statement, lifts the practice far above that platform to the plane of science.

Of course, to homœopathists it will be unsatisfactory, because it is a repetition of the never-ceasing round of empirical prescribing into which regular medicine has degenerated. Still, even the Hahnemannian homœopathist will wish to inform himself of the details of oxygen practice merely as a part of wide knowledge of what is going on in the world, or from curiosity. To such this book is cordially recommended. W. M. J.

